# New Method of Treating Still-born Children

**Published:** 1853-01

**Authors:** T. Wood

**Affiliations:** Cincinnati, Ohio


From the Western Lancet.

*New Method of Treating Still-born Children.* By T. Wood, M. D., of Cincinnati, Ohio.

Mrs. C——— was brought to bed in her first confinement, and had a very protracted and tedious labor, from a rigid and unyielding vulva. The child on delivery was in a state of syncope, so profound as to leave but little hopes for restoration to life. Full five minutes had been lost in fruitless efforts to excite breathing, and the only sign of life in the child was a slight convulsive effort while its lower limbs were yet in the vagina, after which it lay flaccid, ex-sanguineous, and in appearance dead. Cold air, cold water, and brandy had been thrown on its chest without producing the slightest effect, and I was about to inflate its lungs, when I noticed that the vessels of the cord were much distended with blood, and a very feeble pulsation in its arteries. Finding this condition of the cord, suggested the idea that, perhaps, if the blood it contained could be forced into the circulation of the child, it might afford the required stimulation. Instantly acting on the suggestion, I took the cord between my thumb and fingers, and drew its whole length between them, so as to force the blood into the child, when it immediately cried lustily, and animation was completely restored. It had no more difficulty in beginning life, and is now doing well.

I report this case under the impression that this mode of treatment is original.

Since having the above case, I tried the same treatment in a child that was delivered by a long labor embarrassed by convulsions.—Animation was at once restored on forcing the blood from the cord into the circulation of the child, but there was not, previous to resorting to this means, such complete prostration of the child as in the first case, and though effectual, the result was not so striking.

My friend, Dr. A. M. Slocum, informs me that since I related my case to him, he has tried it in a similar prostration of the child, with the same happy result.

